# A dismantling study of comprehensive cognitive remediation for improving employment outcomes: what is the role of computer cognitive training?

**DOI:** 10.1017/S0033291725100986

**Published:** 2025-07-22

**Authors:** Susan R. McGurk, Kim T. Mueser, Haiyi Xie, Philippe Bloch, Nicole R. DeTore, Nicole Pashka, Susan Guarino, Anabelle Ruiz, Clara Elliot, Heather Gagnon, Edward Bailey, Virginia Fraser, Jason Welsh, Harry Cunningham, Lisa Razzano, Rosemarie Wolfe, Robert E. Drake

**Affiliations:** 1 Center for Psychiatric Rehabilitation, Boston University, Boston, MA, USA; 2 Departments of Occupational Therapy and Psychological and Brain Sciences, Boston University, Boston, MA, USA; 3 Department of Biomedical Data Sciences, Geisel School of Medicine at Dartmouth, Hanover, NH, USA; 4 Department of Community and Family Medicine, Geisel School of Medicine at Dartmouth, Hanover, NH, USA; 5 Department of Psychiatry, Massachusetts General Hospital, Boston, MA and Department of Psychiatry, Harvard Medical School, Cambridge, MA, USA; 6 Thresholds, Inc., Chicago, IL, USA; 7 The Mental Health Center of Greater Manchester, Manchester, NH, USA; 8 Department of Psychiatry, University of Illinois, Chicago, IL, USA; 9 Department of Psychiatry, Columbia University, New York, NY, USA

**Keywords:** cognitive remediation, cognitive training, employment, severe mental illness

## Abstract

**Background:**

Comprehensive cognitive remediation improves cognitive and functional outcomes in people with serious mental illness, but the specific components required for effective programs are uncertain. The most common methods to improve cognition are facilitated computerized cognitive training with coaching and teaching cognitive self-management strategies. We compared these methods by dismantling the Thinking Skills for Work program, a comprehensive, validated cognitive remediation program that incorporates both strategies.

**Methods:**

In a randomized controlled trial we assigned 203 unemployed people with serious mental illness in supported employment programs at two mental health agencies to receive either the full Thinking Skills for Work (TSW) program, which included computerized cognitive training (based on Cogpack software), or the program with cognitive self-management (CSM) but no computer training. Outcomes included employment, cognition, and mental health over 2 years. To benchmark outcomes, we also examined competitive work outcomes in a similar prior trial comparing the TSW program with supported employment only.

**Results:**

The TSW and CSM groups improved significantly on all outcomes, but there were no differences between the groups. Competitive work outcomes for both groups resembled those of the TSW program in a prior trial and were better than the supported employment-only group in that study, suggesting that participants in both groups benefited from cognitive remediation.

**Conclusions:**

Providing facilitated computerized cognitive training improved neither employment nor cognitive outcomes beyond teaching cognitive self-management strategies in people receiving supported employment. Computerized cognitive training may not be necessary for cognitive remediation programs to improve cognitive and functional outcomes.

## Introduction

Competitive employment rates in people with serious mental illness are often below 15% (Hakulinen et al., 2020; Marwaha, Durrani, & Singh, 2013; Marwaha & Johnson, 2004), despite most individuals wanting to work (Frounfelker et al., 2011; Mueser, Salyers, & Mueser, 2001; Westcott et al., 2015). The Individual Placement and Support model of supported employment (Becker & Drake, 2003) has been shown to improve work outcomes in this population in numerous controlled studies (Bond et al., 2023; Bond, Drake, & Becker, 2020; Frederick & VanderWeele, 2019). However, not everyone benefits from supported employment; a significant proportion of participants never work (Bond et al., 2020) and many others only work briefly due to negative job endings (Bond & Kukla, 2011; DeTore, Khare, Hintz, & Mueser, 2019; Frederick & VanderWeele, 2019; Teixeira, Mueser, Rogers, & McGurk, 2018). Thus, increasing the effectiveness of supported employment is critical.

Cognitive functioning is an important treatment target for improving work outcomes in supported employment. Impaired cognitive functioning is common in disorders such as schizophrenia (Cooper et al., 2015; Harvey, 2013) and predicts work in both the general population (Richardson & Norgate, 2015; Schmidt & Hunter, 1998) and persons with serious mental illness (Allott et al., 2013; McGurk, Mueser, Mischel, et al., 2013; Tsang et al., 2010), including in people receiving supported employment (McGurk et al., 2018). Cognitive remediation targets abilities such as attention, memory, and executive functions using scientific principles of learning to improve psychosocial adjustment (Wykes, Bowie, & Cella, 2024). Numerous studies have shown that providing cognitive remediation to people receiving vocational rehabilitation improves work outcomes compared to vocational rehabilitation alone (Bell et al., 2005; Bell, Laws, & Petrakis, 2017; Burns & Erickson, 2023; Lindenmayer et al., 2008; McGurk et al., 2015; McGurk et al., 2016; McGurk & Mueser, 2015; McGurk, Mueser, DeRosa, & Wolfe, 2009; McGurk et al., 2007a; Sato et al., 2015; Vauth et al., 2005), although a few studies report no differences (Christensen et al., 2019; Tsang et al., 2016). Nevertheless, the critical ingredients of cognitive remediation programs that improve employment are uncertain.

Cognitive remediation programs employ a variety of strategies and elements to improve individuals’ cognitive abilities and real-world functioning. An expert panel (Bowie et al., 2020) identified four core components of effective programs: (1) engagement with a trained cognitive specialist, (2) repeated practice of cognitive exercises, (3) development of effective cognitive strategies, and (4) facilitation of the transfer of cognitive skills to daily functioning. More effective cognitive strategies can be developed either through coaching during the facilitated practice of cognitive exercises (“strategy coaching”) or by teaching cognitive self-management (e.g. compensatory) strategies. The transfer of cognitive skills involves integrating cognitive remediation with psychosocial interventions (e.g. supported employment) or helping individuals link cognitive skills to their goals.

The most comprehensive cognitive remediation intervention for increasing the effectiveness of vocational services is the Thinking Skills for Work (TSW) program (McGurk & Mueser, 2021), which has improved cognitive and employment outcomes in multiple controlled trials (Burns & Erickson, 2023; Lindenmayer et al., 2008; McGurk et al., 2009; McGurk et al., 2015; McGurk et al., 2016; McGurk & Mueser, 2015; McGurk, Mueser, et al., 2007a; Sato et al., 2015). TSW incorporates all four components recommended by the cognitive remediation expert panel (Bowie et al., 2020), including both approaches to developing cognitive strategies: facilitated computerized cognitive training with strategy coaching and teaching cognitive self-management skills. However, research has not evaluated the unique contribution of these two cognitive remediation methods in the TSW program to improving outcomes. Such research has potential implications for understanding how TSW works and for streamlining the program to improve its efficiency and personalization.

This dismantling study evaluated the benefit of providing facilitated computerized cognitive training in addition to teaching cognitive self-management strategies in the TSW program, compared to teaching self-management strategies alone. We focused on the unique contribution of facilitated computerized cognitive training to TSW (rather than cognitive self-management) for two reasons. First, because computerized cognitive training is more labor-intensive than teaching cognitive self-management strategies, determining its contribution to outcomes has stronger implications for service planning. Second, computerized cognitive training requires investment in equipment, software, specialized training, and so forth, imposing limits on its use, unlike teaching cognitive self-management, underscoring the importance of establishing its benefits to justify its retention in the TSW program.

Our primary hypothesis was that facilitated computerized cognitive training in the TSW program (in addition to teaching cognitive self-management) would be associated with significantly better employment outcomes than teaching cognitive self-management strategies alone, while our secondary hypothesis was that it would also lead to greater improvements in cognitive functioning.

## Methods

A randomized controlled trial was conducted in the supported employment programs of two mental health agencies to compare two cognitive remediation programs based on the TSW program: 1) the “full” TSW program, including facilitated computerized cognitive training and teaching cognitive self-management strategies, and 2) a streamlined version of the program developed for this study, including only teaching cognitive self-management (CSM). All procedures were approved by local institutional review boards and monitored by a data safety and monitoring board.

### Sites

The study sites were two large community mental health centers located in the Northeastern and Midwestern United States. Both centers provide comprehensive psychiatric services for serious mental illness, including pharmacological treatment, case management, and a broad range of psychosocial programs.

### Participants

Inclusion criteria were: (1) meets the state definition of serious mental illness; (2) minimum age 18; (3) fluent in English; (4) enrolled in Individual Placement and Support program; (5) unemployed and wants to work; (6) no evidence of traumatic brain injury or other medical condition with major effect on brain functioning; and (7) cognitively impaired, defined as 1.0 SD below normative scores on either a measure of cognitive flexibility (Radford, Chaney, & O’Leary, 1978) or verbal learning (Brandt, 1991). Deficits in cognitive flexibility and verbal learning were used as inclusion criteria because these cognitive domains are impaired in schizophrenia (Green, Kern, Braff, & Mintz, 2000) and are critical in community functioning, especially employment (McGurk et al., 2003), and are common targets of cognitive remediation (Bowie & Harvey, 2006b).

A total of 203 participants met inclusion criteria, provided informed consent, completed the baseline assessment, and were randomized to either the full TSW program (*n* = 99) or the CSM program (*n* = 104). The CONSORT diagram is provided in Supplemental Figure 1.

### Assessment

Trained clinical interviewers, blind to treatment assignment, assessed neurocognition, symptoms, and quality of life at baseline, and 8, 16, and 24 months later. Prior to each follow-up assessment, participants were instructed not to disclose their group assignment. Inter-rater reliability for symptom and cognitive assessments was established prior to study initiation, with reliability checks conducted on 15% of recorded assessments throughout the study. Participants were paid for completing assessments, but not for involvement in the interventions. Employment was tracked weekly by research assistants through contacts with participants, employment specialists, and treatment team members.

#### Measures

Gender, race, and ethnicity were assessed by self-report. Psychiatric and substance use diagnoses were assessed at baseline with the Structured Clinical Interview for DSM-IV (First, Spitzer, Gibbon, & Williams, 1996) and reading level with the WRAT III Reading Subtest (Wilkinson, 1993). Symptoms were assessed with the Expanded Brief Psychiatric Rating Scale (BPRS; Lukoff, Nuechterlein, & Ventura, 1986), and psychosocial functioning with the Quality of Life Scale (QLS; Heinrichs, Hanlon, & Carpenter, 1984) and the Global Assessment of Functioning (GAF; Jones, Thornicroft, Coffey, & Dunn, 1995). Three questions also asked about participants’ satisfaction with their finances, vocational program, and overall life over the past month; responses ranged from 1 (“Terrible”) to 7 (“Delighted”).

At baseline and all subsequent assessments, interviewers evaluated neurocognition with the Measurement and Treatment Research to Improve Cognition in Schizophrenia Consensus Cognitive Battery (MCCB) (Nuechterlein et al., 2008), with the Overall Composite T-score based on ten MCCB tests used as the primary measure of cognitive functioning. We also administered three additional tests. The learning trials for the Hopkins Verbal Learning Test (HVLT-Sum) and Brief Visual Memory Test (BVMT-Sum) are in the MCCB, but not their corresponding delayed recall trials (HVLT-D, BVMT-D), which assess memory, and thus these two tests were added. The MMCB has only one measure of executive functioning, Mazes, which assesses planning. We added Trails B (Radford et al., 1978) to assess cognitive flexibility and broaden the measurement of executive functioning. Our administration of Trails B extended the discontinuation rule for stopping a participant on the test from 300 to 500 seconds (Heaton, Miller, Taylor, & Grant, 2004) to reduce the floor effect that frequently occurs on this test in schizophrenia (Bowie & Harvey, 2006a; Heinrichs & Zakzanis, 1998) and to broaden the distribution of Trails B scores. We calculated normative scores using Z-scores and converted those to T-scores.

For paid jobs, information was collected on hours and weeks worked, wages earned, job type, job tenure, and whether work was competitive.

### Interventions

All participants were enrolled in high-fidelity Individual Placement and Support programs (Becker & Drake, 2003), as determined by independent expert IPS fidelity evaluators. This included each participant being assigned an employment specialist who provided all their vocational services and received weekly group supervision from a team leader. Participants continued to receive their usual mental health services throughout the study.

#### Thinking Skills for Work (TSW) program

The full TSW program (McGurk & Mueser, 2021) was implemented by cognitive specialists who were members of the supported employment team and coordinated treatment with the employment specialists. TSW includes four phases: (1) assessment, (2) facilitated computerized cognitive training, (3) teaching cognitive self-management strategies, and (4) consultation. Throughout all phases, the cognitive specialist, employment specialist, and participant meet at least monthly to review progress. During the *assessment* phase, the cognitive specialist collaborates with the employment specialist to conduct an employment history with the participant, with a particular focus on recent job losses and the potential role of cognitive difficulties in contributing to them.

*Facilitated computerized cognitive training* is based on a standardized curriculum of exercises selected from COGPACK software (Marker, 2014) shown to improve cognitive functioning in schizophrenia (Sartory, Zorn, Groetzinger, & Windgassen, 2005). The curriculum is organized into 24 lessons of repeated cognitive exercises. The cognitive specialist teaches the participant how to operate the COGPACK software and uses the lesson plans to guide their practice and record their performance. The specialist also links cognitive exercises to work tasks related to the participant’s employment goals, teaches strategies to improve their performance, highlights improvements over time, and encourages and reinforces their effort and persistence during challenging exercises.

*Teaching cognitive self-management* usually begins several weeks or months following the initiation of computerized cognitive training and involves the cognitive specialist helping the participant develop practical strategies for enhancing their cognitive performance in everyday situations (e.g. repeating back verbal information to ensure correct encoding) through modeling, role-playing, and so forth. These sessions are also aimed at harnessing participants’ motivation (and hence effort) to improve their cognitive abilities and achieve their work goals, such as through psychoeducation about the relationship between cognition and work, identifying personal strengths, and challenging negative thinking. The curriculum for teaching cognitive self-management is standardized in ten educational handouts: (1) Cognitive Skills and Work, (2) Recognizing Your Strengths, (3) Challenging Negative Thinking, (4) Improving Attention and Concentration, (5) Reducing Memory Difficulties, (6) Getting Organized at Home, (7) Getting Organized for Your Job Search, (8) Planning Ahead, (9) Solving Problems, and (10) Improving Thinking Speed. Twelve individual or group sessions are recommended to cover the ten topics, although additional sessions are often required for participants to master the material.

The *consultation* phase begins after the participant has completed the computerized cognitive training and cognitive self-management phases and involves monthly meetings between the cognitive specialist, the employment specialist, and the participant to address cognitive and other barriers to getting or maintaining work.

#### Cognitive Self-Management (CSM) program

The CSM program, which was developed for this study, was implemented in an identical fashion as the full TSW program (e.g. assigned a cognitive specialist who worked with the employment specialist), with only one exception: the facilitated computerized cognitive training phase was not provided. The other three phases of the TSW program were provided (i.e. assessment, cognitive self-management, consultation).

#### Staffing of cognitive specialists at study sites

At the Northeast study site serving a relatively rural population, one cognitive specialist provided all of the services individually to participants in both the TSW and CSM programs. At the Midwest study site serving an urban population, two cognitive specialists collaborated in providing the programs. One cognitive specialist provided all the services in the CSM program and taught cognitive self-management strategies to the participants in the TSW program. This cognitive specialist led weekly group sessions to teach the cognitive self-management curriculum to 4–8 participants (from both TSW and CSM programs) per session. Groups were conducted using an open-enrollment format that allowed new participants to join an ongoing group without having to wait for a new group to begin. The ten cognitive self-management topics (and accompanying handouts) were taught over a period of 12 weekly sessions, with the last session immediately followed by a new 12-session cycle of the curriculum; participants could begin the group during any week of the cycle, regardless of the session topic, and remain in the group for 12 (or more) consecutive weeks to cover all the topics. The second cognitive specialist provided all services to participants in the TSW program (primarily facilitation of computerized cognitive training) except for cognitive self-management strategies, which were usually taught in groups by the first specialist (as described above), but were occasionally taught to individuals by the second specialist. The two cognitive specialists worked together and with the employment specialist to coordinate and integrate their services.

#### Definitions of exposure to TSW and CSM programs

Based on previous research (McGurk et al., 2015), the minimum “exposure” to the TSW program to obtain a therapeutic benefit was a priori defined as completing ≥6 computerized cognitive training sessions. Minimum exposure to the CSM program was defined in a parallel fashion as completing ≥6 cognitive self-management sessions.

### Fidelity

Adherence of the cognitive specialists to the teaching methods in the TSW manual (McGurk & Mueser, 2021) was assessed by the first two authors using two fidelity scales completed based on audiotaped sessions: a 15-item scale for computerized cognitive training sessions and a 24-item scale for cognitive self-management sessions. Fidelity ratings based on approximately 15% of all cognitive remediation sessions indicated high adherence of the cognitive specialists to the TSW/CSM programs.

### Screening recruitment and randomization

Supported employment teams identified potentially eligible participants to a research staff member who reviewed the details of the study with them, obtained informed consent, and administered the cognitive screens to confirm cognitive impairment eligibility criteria. Eligible participants were then scheduled for the baseline interview. On completion of the interview, participants were randomized by a computer program to either TSW or CSM, with no study personnel aware of treatment assignments in advance. Randomization was stratified by site, primary diagnosis (schizophrenia-spectrum versus other), and whether the participant was a “nonresponder” to supported employment, defined as enrolled in supported employment for ≥3 months without obtaining competitive work, or being fired or quitting a job that lasted <3 months (McGurk & Mueser, 2015).

### Statistical analysis

#### Statistical power analysis

We reasoned that the benefit associated with providing computerized cognitive training in addition to teaching cognitive self-management strategies (TSW) compared to teaching self-management strategies alone (CSM) would need to be greater than a small effect size to be clinically meaningful and to justify the additional expense of TSW. We therefore powered the sample size to detect the upper range of a small effect size (Cohen, 1992). Statistical power for mixed-effects linear model analyses was estimated based on a longitudinal design with attrition for the major outcome variables specified in the primary and secondary hypotheses (Hedeker, Gibbons, & Waternaux, 1999; Roy, Bhaumik, Aryal, & Gibbons, 2007). We assumed four assessment points, a 0.5 within-subject correlation for work and 0.7 for cognitive functioning, 20% overall attrition rate, and alpha level = 0.05. Using one-tailed significance tests based on the hypothesis that TSW > CSM, an initial sample size of N = 200 resulted in power of 0.80 to detect small effect sizes (*d*) of 0.33 for work and 0.28 for cognition.

#### Comparison of groups at baseline

Demographic, clinical, and cognitive differences between the two groups at baseline were evaluated by conducting *t*-tests or χ^2^ analyses.

### Intent-to-treat analyses

Intent-to-treat statistical analyses were conducted on all outcomes, irrespective of participation in the assigned programs. Changes in cognition, mental health, and satisfaction were evaluated by fitting generalized linear mixed effects models on the respective outcomes (e.g. MCCB composite T-score), with baseline and the 8-, 16-, and 24-month follow-up scores of each variable as the repeated measures, group (TSW or CSM), time, and the group-by-time interaction as the main independent variables, and site, diagnosis, supported employment nonresponder, and education level (< or ≥high school graduate) as a priori defined covariates. For these analyses, the time effect tests whether both groups improved over the study period, and the group-by-time interaction tests differential changes over time between the groups. Group-by-time interaction effects were evaluated with one-tailed significance tests, and the other effects were evaluated with two-tailed significance tests.

Employment outcomes were evaluated in two ways. First, the 2-year follow-up period was divided into 6-month intervals with cumulative work/no work, weeks worked, hours worked, and wages earned aggregated within each interval. General linear mixed models were then fitted for both continuous (with identity link and normal distribution specification for weeks worked, Tweedie distribution for wages earned) and binary (with logit link function and binomial distribution specification) variables (Fitzmaurice, Laird, & Ware, 2011; Hedeker & Gibbons, 2006). The independent variables were group, time, and their interaction, and the same covariates were included as in the cognitive analyses. The main effect of group tests whether one group worked more than the other group, and the group-by-time interaction tests whether one group worked more than the other over time, both of which were evaluated with one-tailed significance tests. Second, employment data were aggregated across the 2-year study period, and the groups were compared using one-tailed Mann–Whitney and χ^2^ tests.

To explore whether participants with a substance use disorder (SUD) differed in their vocational outcomes from those without a SUD, we compared the two groups within each of the TSW and CSM programs on three aggregated competitive employment variables (ever worked, weeks worked, wages earned) using two-tailed χ^2^ and Mann–Whitney tests.

#### Per protocol analyses

Per protocol analyses were conducted on the co-primary outcomes (cognitive functioning and employment) to explore whether similar results were found between the intent-to-treat analyses, including all randomized participants, and the two subgroups of participants who met exposure criteria for their assigned treatment group (see Methods). These analyses included the generalized linear mixed effects models on changes in cognition and the tests on the employment data aggregated over the 2-year study period, as described above.

### Comparison of findings with McGurk et al. (2015) study

As this study compared two active cognitive remediation programs in participants receiving supported employment but did not include a supported employment-only control group, we compared work outcomes for the TSW and CSM groups in this study with our prior randomized controlled trial that compared TSW to Supported Employment Only (McGurk et al., 2015). First, we explored differences between the participants in the two studies (demographics, work history, baseline cognitive and clinical functioning). Second, we compared the cumulative competitive work outcomes of the TSW and CSM groups in the current study with those of the TSW and Supported Employment Only groups in the previous study using two-tailed Mann–Whitney and χ^2^ tests. We chose to use the same statistical tests to compare the cumulative work outcomes between the groups in the two studies as we used to compare these outcomes between the TSW and CSM groups in the current study (rather than statistically control for differences between the studies in participant characteristics) to facilitate a direct comparison of the results between the two sets of analyses.

## Results

No adverse events related to the interventions were reported during the study. The two groups did not differ significantly on any baseline measures (Table 1).

**Table 1.** Participant characteristics by treatment group (*n* = 203)

Characteristic	Cognitive Self-Management Strategies (*n* = 104)		Thinking Skills for Work (*n* = 99)	
*Continuous variables*	*M*	*SD*	*M*	*SD*
Age	43.35	10.67	43.77	10.69
*Categorical variables*	*n*	*%*	*n*	*%*
Biological sex				
Male	57	54.8	65	65.7
Female	47	45.2	34	34.3
Ethnicity				
Hispanic	9	8.7	10	10.1
Non-Hispanic	95	91.3	89	89.9
Race				
White	54	51.9	58	58.6
Black	41	39.4	34	34.3
Asian	0	0	1	1.0
American Indian/Alaska native	1	1.0	1	1.0
More than one race	8	7.7	5	5.1
Completed high school	75	72.1	69	69.7
Ever married	35	33.7	34	34.3
Living independently	65	62.5	60	60.6
Primary psychiatric diagnosis				
Schizophrenia	32	30.7	27	27.3
Schizoaffective disorder	22	21.1	23	23.2
Psychotic disorder nos	1	1.0	1	1.0
Brief psychotic disorder	0	0	1	1.0
Bipolar disorder	12	11.6	9	9.2
Cyclothymia disorder	0	0	1	1.0
Obsessive-compulsive disorder	0	0	1	1.0
Post-traumatic stress disorder	8	7.7	4	4.0
Major depressive disorder	28	26.9	30	30.3
Depressive disorder Nos	1	1.0	2	2.0
Substance use disorder	62	59.6	61	61.6
Competitive employment in the last 5 years	64	61.5	52	52.5
Supported employment nonresponder	56	53.8	52	52.5
Site				
Manchester, NH	40	38.5	37	37.4
Chicago, IL	64	61.5	62	62.6

The 99 participants in TSW completed a mean of 12.6 computerized cognitive training sessions and 7.4 cognitive self-management sessions (20 total sessions), with 62 (63%) meeting the criteria for exposure to the program (≥6 sessions of computerized cognitive training). The 104 participants in CSM completed a mean of 11.8 cognitive self-management sessions, with 75 (72%) meeting exposure criteria (≥6 sessions of cognitive self-management). The subgroup of 62 participants exposed to TSW completed a mean of 19.3 computerized cognitive training sessions and 10.6 cognitive self-management sessions (29.9 total sessions). The subgroup of 75 participants exposed to CSM completed a mean of 15.8 cognitive self-management sessions.

### Cognitive, clinical, and satisfaction outcomes

The statistical analyses comparing changes in cognitive functioning over time between the two groups are summarized in Table 2, with changes in clinical outcomes and satisfaction in Table 3. Cognitive functioning improved from baseline to the follow-ups for both groups, as indicated by significant time effects for the MCCB cognitive composite score and 9 of the 13 tests. Both groups improved similarly over time, with no significant group-by-time interactions. Significant covariate effects indicating lower cognitive functioning across all assessments were found for diagnosis (schizophrenia), education level (<high school), and site (Midwest center), but not supported employment nonresponder status.

**Table 2.** Analyses of cognitive outcomes for intervention groups: Cognitive Self-Management Strategies (CSM) and Thinking Skills for Work (TSW)

Variable	Baseline		8 months		16 months		24 months		Time effect			Group × time interaction		
	*M*	*SD*	*M*	*SD*	*M*	*SD*	*M*	*SD*	*df*	*F*	*p*	*df*	*F*	*p*
Trails, A									487	34.33	<0.001	486	0.62	0.216
CSM	32.85	14.77	36.72	14.82	35.99	15.24	38.48	13.92						
TSW	32.96	13.26	37.24	13.05	37.41	12.83	37.45	13.55						
Trails, B									498	2.62	0.106	488	2.62	0.106
CSM	−15.89	79.36	−15.98	81.62	−6.88	79.71	−20.65	98.76						
TSW	−12.14	77.60	−15.60	91.64	6.95	64.56	0.20	78.51						
Symbol coding									480	0.78	0.377	480	0.56	0.227
CSM	31.68	10.10	32.64	11.53	31.94	12.80	31.91	10.96						
TSW	30.59	7.43	30.99	11.29	31.17	10.23	31.57	10.39						
HVLT-sum									495	8.88	0.003	494	0.39	0.267
CSM	35.11	7.59	35.79	7.60	39.45	10.49	36.37	8.64						
TSW	35.74	7.42	35.72	7.06	38.28	9.09	36.67	7.99						
HVLT- D									489	3.46	0.063	489	0.20	0.329
CSM	30.78	12.33	32.75	13.82	35.71	13.59	31.75	13.76						
TSW	31.63	12.07	32.19	14.07	33.58	13.19	32.86	14.30						
Spatial span									477	3.93	0.048	477	0.25	0.308
CSM	35.57	12.24	37.05	11.75	36.51	10.96	37.85	12.73						
TSW	37.33	12.95	38.84	11.92	38.72	11.74	38.97	12.56						
Letter-number span									482	3.75	0.053	481	0.98	0.161
CSM	34.13	12.79	35.36	12.53	34.93	12.51	34.97	12.28						
TSW	32.87	12.31	33.37	11.71	35.88	11.80	35.19	11.46						
Mazes test									483	12.35	<0.001	482	3.76	0.027
CSM	37.71	8.45	38.69	10.13	39.89	9.02	40.63	9.98						
TSW	36.93	8.06	36.73	7.04	38.28	8.22	37.39	7.18						
BVMT-sum									483	6.00	0.014	482	0.01	0.459
CSM	30.63	12.65	31.97	13.05	31.48	12.74	33.02	13.78						
TSW	30.42	13.07	30.97	11.98	32.20	14.19	32.32	12.43						
BVMT-D									481	5.92	0.015	480	0.44	0.253
CSM	27.77	12.61	30.02	17.29	28.23	16.10	30.10	16.68						
TSW	27.76	16.26	26.72	15.28	28.64	16.82	31.59	16.43						
Category fluency									489	6.71	0.009	488	<0.00	0.493
CSM	37.66	9.47	37.89	9.66	38.68	9.69	39.22	9.81						
TSW	38.29	8.51	38.68	8.93	38.98	9.52	39.42	9.30						
CPT									474	22.33	<0.001	474	1.02	0.156
CSM	34.90	12.99	39.36	13.17	37.16	12.87	37.48	14.86						
TSW	35.14	11.01	36.16	11.96	37.68	11.63	39.10	12.06						
MSCEIT									487	1.35	0.245	487	1.45	0.114
CSM	40.08	12.99	39.25	12.39	39.00	13.48	40.31	13.49						
TSW	39.27	11.33	38.12	12.54	38.98	13.94	36.42	13.37						
MCCB cognitive composite									476	34.49	<0.001	476	0.30	0.293
CSM	35.03	7.21	36.26	8.05	36.50	8.59	37.01	8.51						
TSW	34.95	6.39	35.68	7.35	36.76	7.07	36.45	7.60						

*Note:* Analyses presented are two-tailed tests for time effects and one-tailed tests for interaction effects.

**Table 3.** Analyses of clinical and functional outcomes by intervention group: Cognitive Self-Management Strategies (CSM) and Thinking Skills for Work (TSW)

Variable	Baseline		8 months		16 months		24 months		Time effect			Group × time interaction		
	*M*	*SD*	*M*	*SD*	*M*	*SD*	*M*	*SD*	*df*	*F*	*p*	*df*	*F*	*p*
BPRS total									490	14.22	<0.001	489	0.61	0.435
CSM	48.06	12.36	45.57	11.14	45.41	12.93	43.90	12.50						
TSW	47.44	11.56	45.32	13.54	45.35	16.66	43.82	14.02						
BPRS depression									488	3.09	0.079	488	0.00	0.987
CSM	2.48	0.97	2.38	0.97	2.34	1.01	2.36	1.04						
TSW	2.40	1.10	2.46	1.25	2.41	1.26	2.23	1.08						
BPRS activation									498	16.39	<0.001	498	1.20	0.273
CSM	1.59	0.55	1.41	0.39	1.41	0.43	1.36	0.42						
TSW	1.56	0.63	1.44	0.55	1.41	0.57	1.42	0.56						
BPRS retardation									506	5.08	0.024	506	0.00	0.976
CSM	1.87	0.65	1.87	0.59	1.86	0.67	1.78	0.61						
TSW	1.77	0.50	1.75	0.53	1.78	0.65	1.61	0.54						
BPRS psychosis									493	9.74	0.001	492	1.58	0.209
CSM	2.03	0.98	1.89	0.86	1.86	0.89	1.75	0.85						
TSW	2.08	1.01	1.83	0.91	1.87	1.12	1.85	1.11						
Global Assessment of Functioning									519	47.69	<0.001	518	0.09	0.767
CSM	44.48	8.94	51.44	12.57	51.52	11.83	52.41	12.25						
TSW	43.88	10.23	51.51	13.21	50.52	12.28	51.75	12.81						
QLS Total									491	11.87	<0.001	491	0.84	0.360
CSM	65.43	9.73	69.96	17.54	66.74	17.42	66.14	14.74						
TSW	65.11	14.84	69.94	20.08	67.86	14.20	73.70	15.95						
Life satisfaction									484	9.27	0.002	484	0.02	0.894
CSM	4.50	1.46	4.56	1.46	4.70	1.50	4.82	1.52						
TSW	4.62	1.63	4.88	1.48	4.69	1.50	5.04	1.37						
Satisfaction with finances									484	2.39	0.123	484	4.15	0.042
CSM	3.41	1.69	3.51	1.68	3.42	1.74	3.47	1.64						
TSW	3.49	1.85	3.68	1.76	3.73	1.91	3.87	1.65						
Satisfaction with vocational program									493	2.26	0.133	493	9.13	0.002
CSM	5.60	1.32	5.17	1.42	5.27	1.11	4.94	1.46						
TSW	5.35	1.40	5.38	1.37	5.53	1.30	5.47	1.27						

*Notes:* Analyses presented are two-tailed tests for time effects and one-tailed tests for group-by-time interaction effects.

Abbreviations: BPRS = Brief Psychiatric Rating Scale; QLS = Quality of Life Scale

Clinical and psychosocial functioning outcomes also improved similarly from baseline to the follow-ups for both groups, with significant time effects for the BPRS total and the QLS total, three of the four subscales of the BPRS, and the GAF, but no significant group-by-time interactions. Consistent covariate effects were found, indicating more severe symptomatology on the BPRS and worse functioning on the QLS and GAF for diagnosis (schizophrenia) and site (Northeast), but not for education level or nonresponder status.

Overall satisfaction with life also improved significantly and similarly over time for both groups. However, the groups differed in changes in satisfaction with finances and vocational programs, as indicated by significant group-by-time interactions. Participants in TSW reported greater increases in their satisfaction with these areas than in CSM, as depicted in Supplemental Figure 2. The site covariate for satisfaction with the vocational program was significant, indicating higher satisfaction among participants in the Northeast than the Midwestern site, but none of the other covariates were significant for any of the satisfaction ratings.

### Work outcomes

The linear trend analyses of the cumulative work outcomes over each of the four 6-month periods found no significant group or group-by-time effects, indicating similar outcomes between the TSW and CSM participants over time (Supplemental Table 1). There was a significant time effect indicating increases in wages earned from competitive work and all paid work by both groups over the 2-year study period (*F*s(3) = 15.54, 15.50, *p*s < 0.001, respectively; Supplemental Figure 3), but not for rates of work or weeks worked, indicating relatively stable levels from the first 6-month period to the last 6-month period of the study. Significant covariate effects were found only for diagnosis in the Tweedie models of wages earned; participants with schizophrenia-spectrum disorders earned less total wages and competitive wages over the study period than those with other diagnoses.

The TSW and CSM groups also did not differ significantly on any of the aggregated work outcomes (Table 4). Approximately 68% of the participants obtained competitive work during the study. In the full sample, participants worked an average of 22 weeks and earned about $4000 in competitive wages. Among those who worked competitively, participants worked an average of 33 weeks and earned over $6000.

**Table 4.** Cumulative employment outcomes over 2 years by intervention group: cognitive self-management strategies (CSM) and thinking skills for work (TSW)

Employment measure	Cognitive Self-Management Strategies		Thinking Skills for Work		*Z*	*p*
	*M*	*SD*	*M*	*SD*		
Competitive employment						
Number of jobs worked	1.51	1.88	1.08	1.20	−1.063	0.144
Weeks worked	24.01	29.25	21.00	28.63	−0.877	0.190
Waged earned ($)	3,959.69	5,702.83	4,043.38	7,360.88	−0.782	0.217
Hours worked	421.71	593.20	404.25	650.15	−0.804	0.211
Length of first job (weeks)	28.29	22.96	27.73	27.51	−0.789	0.215
All paid employment						
Number of jobs worked	1.73	0.933	1.23	1.35	−1.110	0.134
Weeks worked	25.79	30.77	22.72	20.16	−0.359	0.360
Waged earned ($)	3,959.69	5,702.83	4,043.38	7,360.88	−0.779	0.218
Hours worked	421.71	593.20	419.29	646.74	−0.789	0.215
Length of first job (weeks)	32.34	24.41	29.77	28.40	−1.120	0.132
	N	%	N	%	χ*^2^*	*p*
Any competitive work	73	70.2	66	66.7	0.292	0.295
Any work	75	72.1	70	70.7	0.049	0.412

*Notes: Z* statistic using Mann–Whitney test and χ*^2^* statistic using Walk χ*^2^* test.

All analyses are one-tailed tests.

Comparisons of participants with a SUD to those without a SUD on the three selected aggregated competitive employment outcomes revealed no significant differences in any outcomes between the two groups within either the TSW or CSM program (*p*s > 0.1). In TSW, 33/61 (55.1%) of participants with a SUD worked versus 23/38 (60.5%) of those with no SUD, with 22.11 (*SD* = 28.89) versus 16.98 (*SD* = 26.60) weeks worked and $3,182.40 (*SD* = 4,494.73) versus $3,832.38 (*SD* = 7,993.60) wages earned, respectively. In CSM, 41/62 (66.1%) of participants with a SUD worked versus 25/42 (59.5%) of those with no SUD, with 20.17 (*SD* = 26.10) versus 25.06 (*SD* = 30.33) weeks worked and $3,444.54 (*SD* = 5,689.76) versus $4,395.22 (*SD* = 5,689.76) wages earned, respectively.

### Per protocol analyses

The results of the mixed effects linear regression analyses comparing changes in cognitive functioning over time between the subgroups of participants who were exposed to the TSW program (*n* = 62) or the CSM program (*n* = 75) are summarized in Supplemental Table 2. The findings were similar to the intent-to-treat analyses, with participants in TSW not showing greater improvements than those in CSM on any of the cognitive measures.

The analyses comparing the exposed TSW and CSM groups on cumulative employment outcomes over the two-year study period are summarized in Supplemental Table 3. The findings were also similar to the intent-to-treat analyses and indicated no significant differences between the two groups on any of the employment outcomes.

### Comparison with McGurk et al. (2015) study

There were several significant differences between the participants in the current dismantling study and those in the McGurk et al. (2015) study. A higher percentage of participants in the dismantling study were Black (37%) than in McGurk et al. (2015) (10%), and a lower percentage were nonresponders to supported employment (100% versus 53%, respectively) (Supplemental Table 4). (Note that nonresponder status was not significantly related to work outcomes in the current dismantling study.) Participants in the current study also had significantly greater illness severity than those in McGurk et al. (2015), including worse cognitive functioning, symptoms, and psychosocial functioning (Supplemental Table 5).

**Figure 1.**
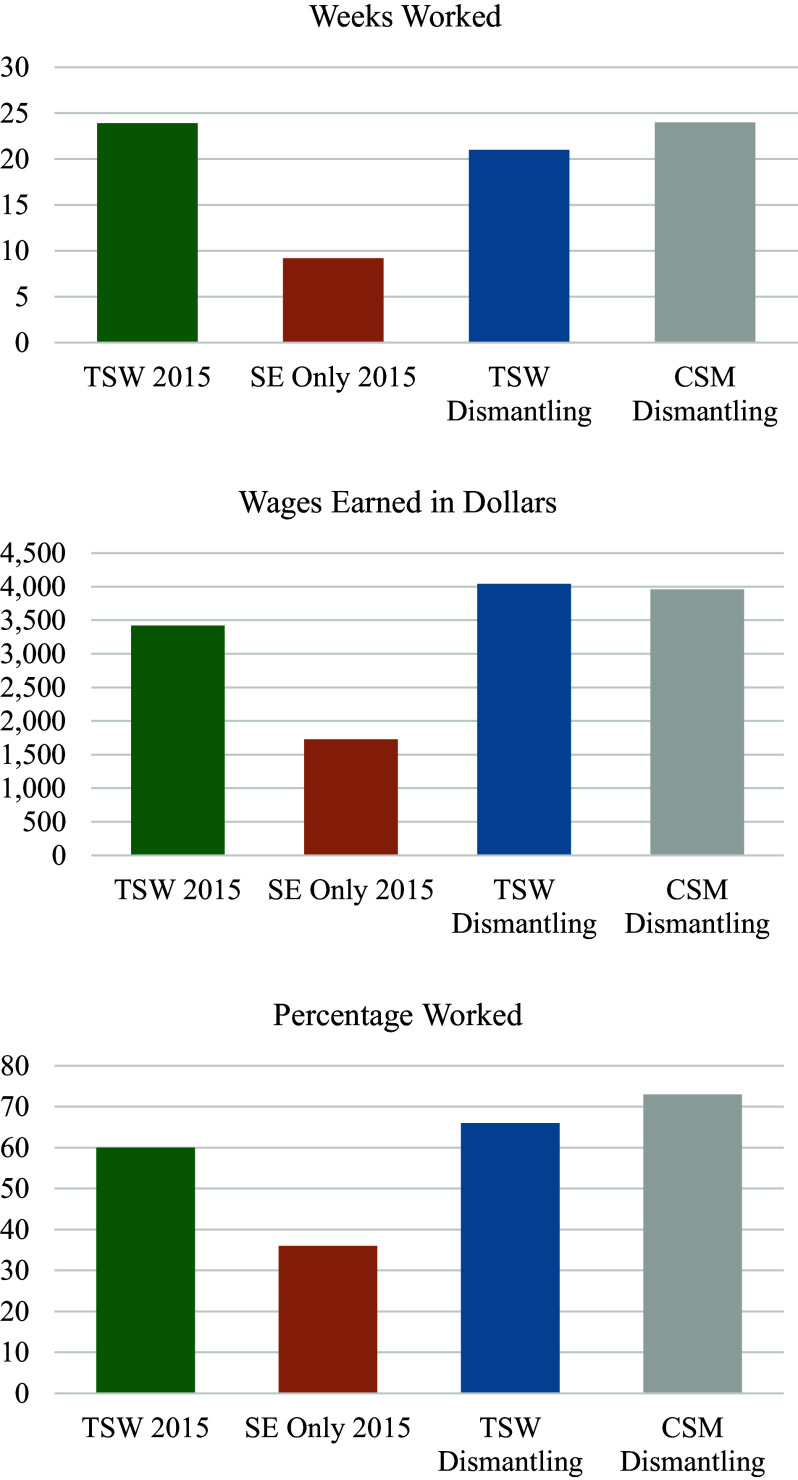
Cumulative 2-year work outcomes for treatment groups in McGurk et al. (2015) study and current dismantling study by intervention group. Notes: CSM, Cognitive Self-Management Strategies program; SE Only, supported employment only; TSW, Thinking Skills for Work program. Across all three cumulative work outcomes (weeks worked, wages earned, percentage worked), the TSW 2015, TSW Dismantling, and CSM Dismantling groups were comparable (not statistically different), whereas all three groups had significantly better outcomes than the SE Only 2015 group; see Supplemental Table 6.

Comparing the cumulative work outcomes (ever worked, hours worked, wages earned) of the treatment groups in the current dismantling study (TSW and CSM) with the treatment groups in McGurk et al. (2015) (TSW and Supported Employment Only) revealed a consistent pattern across all three work variables (Figure 1). Participants in both the TSW and CSM groups in the current study had similar competitive work outcomes to those in the TSW group in McGurk et al. (2015) but had significantly better outcomes than those in the Supported Employment Only group in the 2015 study (Supplemental Table 6). For example, only 36% of the Supported Employment Only group in McGurk et al. (2015) worked competitively, compared to 60% of TSW in that study, 67% of TSW in the current study, and 70% of CSM in the current study.

## Discussion

This study sought to “dismantle” the comprehensive TSW program, which incorporates two approaches to improving cognition (facilitated computerized cognitive training and teaching cognitive self-management strategies), by evaluating the unique benefit of computerized cognitive training in the full program compared to a streamlined version of the program including only cognitive self-management (CSM). We hypothesized that the combination of computerized cognitive training and teaching self-management strategies in the TSW program would lead to better cognitive and employment outcomes than teaching self-management strategies alone in the CSM program. However, in the intent-to-treat analyses, participants in both groups improved similarly in cognitive functioning over the study period and did not differ in their employment outcomes, indicating that facilitated computerized cognitive training provided no additional benefit beyond just teaching cognitive self-management strategies alone. Similar results were found when per-protocol analyses were conducted on the subgroups of participants in the two groups who met minimal exposure criteria to their assigned intervention, suggesting the lack of differences in outcomes between the groups cannot be explained by an insufficient number of computerized cognitive training sessions in the TSW group.

Although the TSW and CSM programs did not differ in cognitive and vocational functioning outcomes, there is evidence that both programs improved these outcomes beyond the effects of supported employment alone. Regarding cognition, prior research has shown that adding TSW to vocational services improves cognitive functioning compared to continued vocational services alone, where few gains have been observed (Lindenmayer et al., 2008; McGurk et al., 2009; McGurk et al., 2015; McGurk et al., 2016; McGurk & Mueser, 2015; McGurk, Mueser, et al., 2007a; Sato et al., 2015). The significant improvements in performance on the MCCB for participants in the TSW and CSM programs are unlikely due to practice effects, as negligible practice effects have been reported for the MCCB over briefer follow-up periods (Buchanan et al., 2011; Keefe et al., 2011), suggesting these gains in cognitive functioning reflect treatment effects of both programs.

Regarding employment, previous studies have also consistently found that adding the TSW program to vocational services improves work outcomes compared to continued vocational services alone (Burns & Erickson, 2023; Lindenmayer et al., 2008; McGurk et al., 2009; McGurk et al., 2015; McGurk et al., 2016; McGurk & Mueser, 2015; McGurk, Mueser, et al., 2007a; Sato et al., 2015), suggesting that the strong implementation of TSW in the present study may have produced similar benefits at work. Additional evidence for the effectiveness of both the TSW and CSM programs at improving vocational functioning is provided by comparing the work outcomes of these two groups with those from a prior randomized controlled trial (McGurk et al., 2015) that compared TSW with Supported Employment Only and was conducted at the same study sites, in the same supported employment programs, and using the same research methods. The cumulative competitive work outcomes over 2 years of both the TSW and CSM groups in the current study were comparable to those of the TSW group in the prior study (McGurk et al., 2015), and significantly better than the Supported Employment Only group in that study (Figure 1). Furthermore, the employment outcomes of the TSW and CSM programs in this study were similar to those in the TSW program in McGurk et al. (2015) despite the fact that participants in this study had significantly worse cognitive functioning than those in the prior study, an important predictor of work outcomes in supported employment (Bergdolt et al., 2022; Mahmood et al., 2019; McGurk et al., 2003; McGurk et al., 2018; Reddy & Kern, 2014). Together, these findings suggest that the TSW and CSM programs in the current dismantling study improved work outcomes more than would be expected from supported employment alone.

The absence of beneficial effects associated with providing facilitated computerized cognitive training in addition to teaching cognitive self-management strategies is striking, considering that repeated practice of cognitive exercises has long been regarded as a critical feature of cognitive remediation programs (McGurk et al., 2007b; Vita et al., 2021; Wykes et al., 2011). However, the evidence supporting this assumption is not strong, and few studies have directly examined it. Although the cognitive remediation expert panel (Bowie et al., 2020) recommended four core elements of effective programs (engagement with a trained therapist, repeated practice of cognitive exercises, development of cognitive strategies, facilitation of transfer of cognitive skills to daily functioning), a recent meta-analysis failed to find that studies that included practice of cognitive exercises had better cognitive or other outcomes than those that did not (Vita et al., 2021).

Cognitive remediation researchers have historically distinguished between “restorative” approaches to improving cognitive functioning, which have been assumed to require practice of cognitive exercises often paired with teaching more effective strategies, and “compensatory” approaches aimed at reducing functional problems by teaching practical cognitive self-management strategies (Bowie et al., 2020; McGurk, Mueser, Covell, et al., 2013). The present findings challenge these assumptions in two ways. First, they showed that teaching compensatory (or self-management) strategies alone does more than compensate for cognitive difficulties, and that it in fact improves neuropsychological test performance, as has been reported by other researchers (Dark et al., 2022; Twamley et al., 2012), and thus has restorative effects on cognitive functioning. Second, the lack of incremental benefit of practice of computerized cognitive exercises in addition to teaching cognitive self-management strategies suggests that, contrary to assumptions, repeated practice of cognitive exercises may not be necessary to improve cognitive functioning or functional outcomes in cognitive remediation programs, in line with Vita et al.’s (2021) meta-analysis.

Twenty to 40 hours of repeated practice of computer cognitive exercises, frequently facilitated by a therapist, is common in cognitive remediation programs (Bowie et al., 2020). Although engaging participants in cognitive exercises may provide opportunities for teaching more effective cognitive strategies, it may be an inefficient use of time if practicing the exercises themselves confers no additional benefit. While replication of these findings is needed, further research should evaluate whether equally effective but less intensive cognitive remediation programs can be developed by reducing time spent on cognitive exercises and increasing focus on directly enhancing the effectiveness of participants’ cognitive strategies.

An intriguing finding was that participants in TSW reported significantly greater increases in satisfaction with their vocational program and their finances over the 2 years than participants in the CSW program. These unexpected differences in satisfaction occurred despite comparable increases in employment between the two groups, including in wages earned. As both programs were integrated with supported employment, the higher satisfaction of the TSW program participants appears to reflect the impact of receiving computerized cognitive training. It is unclear why this specific component of TSW led to the higher satisfaction ratings. Cognitive training in TSW involves the cognitive specialist teaching participants how to operate the Cogpack software package, how to follow the specific curriculum of cognitive exercises and track their performance scores, and strategies for improving performance on the exercises, which are combined with linking exercises to work goals and abundant encouragement and reinforcement of effort and progress on exercises (McGurk & Mueser, 2021). It is possible that learning how to operate the Cogpack software and seeing one’s performance on the cognitive exercises improve over brief periods of time with practice and trying new strategies was a more immediately rewarding experience than learning cognitive self-management strategies, which required more time and effort to produce positive effects in the long run, resulting in higher satisfaction with the TSW program despite its greater length.

Since the development of Cogpack software in 1987 and over subsequent updates of the program, it has been shown to improve cognitive functioning in numerous randomized controlled trials, including when added to vocational rehabilitation (Lindenmayer et al., 2008; McGurk et al., 2009; McGurk et al., 2015; McGurk et al., 2016; McGurk, Mueser, & Pascaris, 2005; Sato et al., 2014; Sato et al., 2015), when added to another psychosocial program (Bernabei et al., 2020; Caponnetto et al., 2018; Cavallaro et al., 2009; Iwata et al., 2017; Lindenmayer et al., 2013), and when provided as a stand-alone treatment (Hatami et al., 2021; Montemagni et al., 2021; Sartory et al., 2005). Numerous other cognitive remediation software packages have been developed since Cogpack, and the field has actively debated the critical elements of programs that optimize neuroplastic learning, raising the question of the generalizability of the findings in this study for Cogpack to other cognitive training packages. However, this debate has been ongoing for decades without reaching a consensus, and there is no clear evidence establishing the superiority of one program over another or of the critical methods, design, or content of cognitive training software programs necessary for enhancing cognition. Considering that Cogpack has considerably more empirical support for improving cognitive functioning than any other program, the principle of Occam’s razor would suggest one assume that the findings reported here for Cogpack are generalizable to other cognitive training software packages until evidence to the contrary becomes available.

The results from the present study suggest that the comprehensive TSW program, which includes both facilitated computerized cognitive training and teaching cognitive self-management strategies, can be more efficiently delivered by just focusing on cognitive self-management, without the loss of any cognitive or employment benefits. Considering the strong evidence supporting the effectiveness of TSW for improving work outcomes in people receiving supported employment, a briefer version of the program could facilitate its implementation by reducing the time required to provide it, expenses related to computerized cognitive training (e.g. computers, software licenses), and opportunity costs for participants. Research is needed to increase access to the streamlined TSW program and further improve the effectiveness of supported employment and competitive work outcomes.

## Supporting information

McGurk et al. supplementary material McGurk et al. supplementary material

## Supplementary material

The supplementary material for this article can be found at http://doi.org/10.1017/S0033291725100986.
