# A Dismantling Study of Comprehensive Cognitive Remediation for Improving Employment Outcomes: What is the Role of Computer Cognitive Training? – CORRIGENDUM

**DOI:** 10.1017/S0033291725101487

**Published:** 2025-08-15

**Authors:** Susan R. McGurk, Kim T. Mueser, Haiyi Xie, Philippe Bloch, Nicole R. DeTore, Nicole Pashka, Susan Guarino, Anabelle Ruiz, Clara Elliot, Heather Gagnon, Edward Bailey, Virginia Fraser, Jason Welsh, Harry Cunningham, Lisa Razzano, Rosemarie Wolfe, Robert E. Drake

**Affiliations:** 1 Center for Psychiatric Rehabilitation, Boston University, Boston, MA, USA; 2 Departments of Occupational Therapy and Psychological and Brain Sciences, Boston University, Boston, MA, USA; 3 Department of Biomedical Data Sciences, Geisel School of Medicine at Dartmouth, Hanover, NH, USA; 4 Department of Community and Family Medicine, Geisel School of Medicine at Dartmouth, Hanover, NH, USA; 5 Department of Psychiatry, Massachusetts General Hospital, Boston, MA and Department of Psychiatry, Harvard Medical School, Cambridge, MA, USA; 6 Thresholds, Inc., Chicago, IL, USA; 7 The Mental Health Center of Greater Manchester, Manchester, NH, USA; 8 Department of Psychiatry, University of Illinois, Chicago, IL, USA; 9 Department of Psychiatry, Columbia University, New York, NY, USA

When this article was originally published in Psychological Medicine it contained an error in the name of the author Lisa Razzano. This has been updated in the original article.

The authors apologise for this error.
