# Supplementary material for: A dismantling study of comprehensive cognitive remediation for improving employment outcomes: what is the role of computer cognitive training?
Source: Psychol Med. 2025 Jul 22;55:e208. doi: 10.1017/S0033291725100986 (PMC12315654; doi:10.1017/S0033291725100986)
Supplement: McGurk et al. supplementary material [file S0033291725100986sup001.docx]

Supplemental Table 1. Results of Linear Regression Analyses of Work Outcomes Per 6-Month Intervals Over 2-Year Study Period by Intervention Group: Cognitive Self-Management (CSM) and Thinking Skills for Work (TSW)

Notes: Time effects are two-tailed tests and treatment group effects are one-tailed tests.

No group by time interaction effects were significant.

|  | Time Effect | | | Treatment Group Effect | | |
| --- | --- | --- | --- | --- | --- | --- |
| Work Variable | *F* | *df* | *p* | *F* | *df* | *p* |
| Any competitive work | .78 | 3 | .506 | .15 | 1 | .350 |
| Weeks of competitive work | 1.26 | 3 | .287 | .84 | 1 | .181 |
| Wages from competitive work | 15.540 | 3 | .001 | .137 | 1 | .356 |
| Any paid work | .39 | 3 | .759 | .04 | 1 | .424 |
| Weeks of paid work | .96 | 3 | .411 | .49 | 1 | .242 |
| Wages from paid work | 15.500 | 3 | .001 | .135 | 1 | .357 |

Supplemental Table 2. Per Protocol Analyses of Cognitive Outcomes by Intervention Group: Cognitive Self-Management Strategies (CSM) and Thinking Skills for Work (TSW) in Participants Exposed to Each Intervention

|  | Baseline | | 8 Month | | 16 Months | | 24 Month | | | Time Effect | | | | | | Group x Time Interaction | | | | | | |  |  |
| --- | --- | --- | --- | --- | --- | --- | --- | --- | --- | --- | --- | --- | --- | --- | --- | --- | --- | --- | --- | --- | --- | --- | --- | --- |
| Variable | *M* | *SD* | *M* | *SD* | *M* | *SD* | *M* | *SD* | | *df* | | *F* | | *p* | | | *df* | | *F* | | | *p* | |  |
| Trails, A |  |  |  |  |  |  |  |  | | 355 | | 33.79 | | <.001 | | | 355 | | 1.23 | | | .134 | |  |
| CSM | 31.76 | 14.97 | 36.35 | 15.12 | 35.47 | 15.92 | 39.13 | 13.99 | |  | |  | |  | | |  | |  | | |  | |  |
| TSW | 30.71 | 13.88 | 36.22 | 14.28 | 37.51 | 12.17 | 35.62 | 13.68 | |  | |  | |  | | |  | |  | | |  | |  |
| Trails, B |  |  |  |  |  |  |  |  | | 360 | | .63 | | .428 | | | 360 | | 2.13 | | | .073 | |  |
| CSM | -12.38 | 69.21 | -6.67 | 67.21 | -7.28 | 79.25 | -19.78 | | 97.83 | |  | |  | |  | | |  | |  |  | | | |
| TSW | -12.47 | 78.93 | -7.10 | 88.22 | 9.21 | 64.18 | -5.54 | | 88.67 | |  | |  | |  | | |  | |  |  | | | |
| Symbol Coding | | |  |  |  |  |  | |  | | 352 | | .39 | | .534 | | | 352 | | .82 | .183 | | | |
| CSM | 32.35 | 10.62 | 33.51 | 11.61 | 32.85 | 13.34 | 31.95 | | 11.85 | |  | |  | |  | | |  | |  |  | | | |
| TSW | 29.92 | 10.46 | 30.53 | 11.06 | 31.30 | 10.05 | 30.67 | | 10.39 | |  | |  | |  | | |  | |  |  | | | |
| HVLT-Sum | |  |  |  |  |  |  | |  | | 358 | | 10.35 | | .001 | | | 357 | | .60 | .220 | | | |
| CSM | 35.68 | 7.25 | 35.35 | 7.54 | 39.39 | 11.35 | 36.29 | | 8.59 | |  | |  | |  | | |  | |  |  | | | |
| TSW | 35.89 | 7.92 | 35.41 | 6.84 | 38.98 | 9.56 | 36.73 | | 7.77 | |  | |  | |  | | |  | |  |  | | | |
| HVLT- D |  |  |  |  |  |  |  |  | | 353 | | 3.56 | | .060 | | | 353 | | .28 | | | .298 | |  |
| CSM | 31.21 | 11.99 | 32.73 | 14.07 | 36.10 | 14.19 | 32.13 | 14.58 | |  | |  | |  | | |  | |  | | |  | |  |
| TSW | 31.34 | 13.12 | 31.90 | 13.98 | 34.71 | 13.29 | 33.25 | 14.25 | |  | |  | |  | | |  | |  | | |  | |  |
| Spatial Span | |  |  |  |  |  |  |  | | 351 | | 2.47 | | .117 | | | 351 | | 1.29 | | | .128 | |  |
| CSM | 36.60 | 11.89 | 37.48 | 12.09 | 36.75 | 10.97 | 39.23 | 12.99 | |  | |  | |  | | |  | |  | | |  | |  |
| TSW | 38.63 | 12.73 | 38.23 | 11.67 | 40.00 | 11.36 | 38.57 | 12.29 | |  | |  | |  | | |  | |  | | |  | |  |
| Letter-Number Span | | | |  |  |  |  |  | | 354 | | 1.75 | | .187 | | | 354 | | .89 | | | .173 | |  |
| CSM | 35.24 | 12.79 | 36.42 | 12.27 | 34.98 | 12.72 | 36.01 | 12.36 | |  | |  | |  | | |  | |  | | |  | |  |
| TSW | 34.66 | 12.68 | 32.71 | 11.87 | 38.15 | 11.87 | 35.78 | 11.89 | |  | |  | |  | | |  | |  | | |  | |  |
| Mazes Test | |  |  |  |  |  |  |  | | 350 | | 11.22 | | <.001 | | | 350 | | 3.38 | | | .033 | |  |
| CSM | 38.31 | 8.81 | 39.96 | 10.69 | 40.39 | 9.02 | 41.92 | 10.20 | |  | |  | |  | | |  | |  | | |  | |  |
| TSW | 37.24 | 8.53 | 37.06 | 7.51 | 38.36 | 8.20 | 37.24 | 7.44 | |  | |  | |  | | |  | |  | | |  | |  |
| BVMT- Sum | |  |  |  |  |  |  |  | | 354 | | 5.98 | | .015 | | | 354 | | .01 | | | .489 | |  |
| CSM | 31.39 | 12.94 | 32.92 | 13.31 | 32.50 | 12.86 | 33.89 | 14.54 | |  | |  | |  | | |  | |  | | |  | |  |
| TSW | 31.03 | 13.83 | 30.08 | 12.90 | 31.88 | 14.09 | 33.02 | 13.20 | |  | |  | |  | | |  | |  | | |  | |  |
| BVMT-D |  |  |  |  |  |  |  |  | | 352 | | 7.98 | | .005 | | | 352 | | .04 | | | .425 | |  |
| CSM | 28.32 | 17.05 | 30.73 | 17.17 | 29.73 | 16.54 | 31.48 | 16.97 | |  | |  | |  | | |  | |  | | |  | |  |
| TSW | 28.03 | 16.94 | 26.00 | 16.24 | 27.83 | 16.86 | 32.20 | 17.70 | |  | |  | |  | | |  | |  | | |  | |  |
| Category Fluency | | |  |  |  |  |  |  | | 357 | | 6.52 | | .011 | | | 356 | | .81 | | | .184 | |  |
| CSM | 37.11 | 9.96 | 37.76 | 9.35 | 38.42 | 9.86 | 39.56 | 10.10 | |  | |  | |  | | |  | |  | | |  | |  |
| TSW | 37.79 | 8.50 | 38.39 | 8.50 | 38.50 | 9.17 | 38.73 | 9.21 | |  | |  | |  | | |  | |  | | |  | |  |
| CPT |  |  |  |  |  |  |  |  | | 349 | | 20.92 | | <.001 | | | 349 | | .68 | | | .204 | |  |
| CSM | 35.53 | 12.98 | 37.84 | 13.58 | 37.03 | 13.11 | 38.71 | 14.45 | |  | |  | |  | | |  | |  | | |  | |  |
| TSW | 34.68 | 11.33 | 36.82 | 12.36 | 37.98 | 12.10 | 39.18 | 13.32 | |  | |  | |  | | |  | |  | | |  | |  |
| MSCEIT |  |  |  |  |  |  |  |  | | 354 | | .88 | | .349 | | | 353 | | 3.45 | | | .032 | |  |
| CSM | 40.44 | 13.09 | 40.19 | 12.76 | 39.82 | 14.21 | 41.60 | 13.15 | |  | |  | |  | | |  | |  | | |  | |  |
| TSW | 38.71 | 12.04 | 37.47 | 12.96 | 37.90 | 14.53 | 35.92 | 13.13 | |  | |  | |  | | |  | |  | | |  | |  |
| MCCB Cognitive Composite | | | |  |  |  |  |  | | 351 | | 35.27 | | <.001 | | | 351 | | 1.35 | | | .123 | |  |
| CSM | 35.34 | 7.46 | 36.76 | 8.48 | 36.78 | 9.13 | 37.81 | 8.91 | |  | |  | |  | | |  | |  | | |  | |  |
| TSW | 34.93 | 7.02 | 35.28 | 7.70 | 37.04 | 6.92 | 36.15 | 7.69 | |  | |  | |  | | |  | |  | | |  | |  |

Note: Analyses presented are two-tailed tests for time effects and one-tailed tests for group by time interaction effects.

Supplemental Table 3. Per Protocol Analyses of Cumulative Employment Outcomes Over 2 Years by Intervention Group: Cognitive Self-Management Strategies (CSM) and Thinking Skills for Work (TSW) in Participants Exposed to Each Intervention

| Employment Measure | Cognitive Self-Management Strategies  (n = 75) | | Thinking Skills for Work  (n = 62) | |  | |
| --- | --- | --- | --- | --- | --- | --- |
|  | *M* | *SD* | *M* | *SD* | *Z* | *p* |
| Competitive Employment |  |  |  |  |  |  |
| Number of jobs worked | 1.43 | 1.66 | 1.13 | 1.14 | -.557 | .578 |
| Weeks worked | 22.73 | 28.6 | 25.29 | 31.35 | -.179 | .858 |
| Waged earned ($) | 3,781.37 | 5,610.81 | 4,714.51 | 8,039.99 | -.084 | .933 |
| Hours worked | 409.16 | 597.65 | 468.20 | 699.65 | -.075 | .940 |
| Length of first job (weeks) | 27.69 | 22.92 | 34.21 | 25.10 | -.819 | .413 |
| All Paid Employment |  |  |  |  |  |  |
| Number of jobs worked | 1.65 | 1.89 | 1.32 | 1.29 | -.472 | .637 |
| Weeks worked | 27.69 | 31.48 | 28.98 | 32.50 | -.253 | .802 |
| Waged earned ($) | 3,781.37 | 5,610.81 | 4,717.34 | 8,036.94 | -.089 | .929 |
| Hours worked | 438.73 | 599.63 | 483.31 | 694.66 | -.121 | .904 |
| Length of first job (weeks) | 32.61 | 25.10 | 36.05 | 30.54 | -.361 | .718 |
|  |  |  |  |  |  |  |
|  | N | % | N | % | χ^2^ | *p* |
| Any competitive work | 49 | 65.3 | 39 | 62.9 | .087 | .768 |
| Any work | 51 | 68.0 | 43 | 69.4 | .029 | .865 |

Notes: *Z* statistic using Mann-Whitney test and χ*^2^* statistic using Walk χ*^2^* test.

All analyses presented are one-tailed tests.

Supplemental Table 4. Differences in Participant Characteristics between McGurk et al. (2015) Study and Current Dismantling Study

| Characteristic | 2015 (n = 107) | | Dismantling (n = 203) | | Analysis | |
| --- | --- | --- | --- | --- | --- | --- |
|  | *M* | *SD* | *M* | *SD* | *t* | *p* |
| Age | 44.15 | 11.06 | 43.56 | 10.65 | 0.42 | .672 |
|  |  |  |  |  |  |  |
|  | *n* | *%* | *n* | *%* | χ^2^ | *p* |
| Biological Sex |  |  |  |  | 0.84 | .359 |
| Male | 70 | 65.4 | 122 | 60.1 |  |  |
| Female | 37 | 34.6 | 81 | 39.9 |  |  |
| Ethnicity |  |  |  |  | 0.27 | .605 |
| Hispanic | 12 | 11.2 | 19 | 9.4 |  |  |
| Non-Hispanic | 95 | 88.8 | 184 | 90.6 |  |  |
| Race |  |  |  |  | 5.69 | <.001 |
| White | 92 | 86.0 | 112 | 55.2 |  |  |
| Black | 11 | 10.3 | 75 | 36.9 |  |  |
| Asian | 2 | 1.9 | 1 | 0.5 |  |  |
| American Indian/ Alaska Native | 0 | 0 | 2 | 1.0 |  |  |
| More than One Race | 2 | 1.9 | 13 | 6.4 |  |  |
| Completed High School | 76 | 71.0 | 144 | 70.9 | 0.02 | .987 |
| Ever Married | 38 | 35.5 | 69 | 34.0 | 0.27 | .789 |
| Living Independently | 63 | 61.2 | 125 | 61.6 | 0.01 | .944 |
| Schizophrenia Diagnosis | 49 | 45.8 | 104 | 51.2 | 0.91 | .364 |
| Substance Use Diagnosis | 31 | 29.2 | 41 | 20.2 | 0.03 | .866 |
| Competitive Employment in Last 5 Years | 68 | 63.7 | 116 | 57.1 | 1.19 | .274 |

Note: All analyses presented are two-tailed tests.

Supplemental Table 5. Baseline Cognitive, Symptom, and Functional Differences between McGurk et al. (2015) Study and the Current Dismantling Study

| Measure | 2015 (n = 107) | | Dismantling (n = 203) | | Analysis | |
| --- | --- | --- | --- | --- | --- | --- |
|  | *M* | *SD* | *M* | *SD* | *t* | *p* |
| Cognitive Battery |  |  |  |  |  |  |
| Trails A | 36.74 | 12.67 | 32.91 | 14.02 | 2.36 | .019 |
| Trails B | 24.76 | 16.94 | -14.07 | 78.33 | 4.95 | <.001 |
| BACS | 32.21 | 10.65 | 31.15 | 10.16 | 0.86 | .392 |
| HVLT Sum | 36.69 | 9.16 | 35.42 | 7.50 | 1.31 | .190 |
| HVLT Delayed | 34.24 | 13.29 | 31.19 | 12.18 | 2.02 | .044 |
| Spatial SPAN | 38.51 | 12.83 | 36.43 | 12.60 | 1.38 | .169 |
| Letter Number Sequence | 37.74 | 13.65 | 33.52 | 12.54 | 2.73 | .007 |
| Mazes | 38.73 | 9.21 | 37.33 | 8.25 | 1.36 | .174 |
| BVMT Sum | 34.43 | 13.63 | 30.53 | 12.83 | 1.49 | .014 |
| BVMT Delayed | 33.52 | 16.10 | 27.76 | 16.40 | 2.95 | .003 |
| Fluency | 39.28 | 10.13 | 37.97 | 9.00 | 1.17 | .245 |
| MSCEIT | 39.95 | 12.52 | 39.69 | 12.19 | 0.18 | .858 |
| CPT- Mean | 33.75 | 11.78 | 35.02 | 11.92 | -0.89 | .376 |
| MCCB Cognitive Composite | 37.22 | 8.14 | 35.00 | 6.81 | 2.55 | .011 |
| PANSS/BPRS* |  |  |  |  |  |  |
| Positive | 2.02 | 0.88 | 2.02 | 0.88 | -1.18 | .238 |
| Negative | 2.19 | 1.01 | 2.27 | 1.04 | -0.56 | .574 |
| Disorganization | 1.53 | 0.95 | 1.95 | 1.32 | -2.70 | .007 |
| Excitement | 1.38 | 0.53 | 1.73 | 0.75 | -3.95 | <.001 |
| Depression | 2.80 | 1.19 | 2.73 | 1.37 | 0.38 | .702 |
| QLS |  |  |  |  |  |  |
| Interpersonal Relations | 3.07 | 1.34 | 2.46 | 1.13 | 4.18 | <.001 |
| Instrumental Role | 2.76 | 0.80 | 1.20 | 0.67 | 18.27 | <.001 |
| Intrapsychic  Foundations | 3.75 | 0.95 | 2.84 | 0.95 | 7.94 | <.001 |
| Common Objects and  Activities | 2.85 | 0.88 | 3.97 | 0.99 | -1.02 | .309 |
| GAF | 46.97 | 8.51 | 44.19 | 9.57 | 2.34 | .200 |

Notes: All analyses presented are two-tailed tests.

BPRS = Brief Psychiatric Rating Scale; GAF = Global Assessment of Functioning; PANSS = Positive and Negative Syndrome Scale; QLS = Quality of Life Scale

*The PANSS and BPRS overlap on 16 items; therefore, those 16 items were averaged to create comparable subscales (DeTore et al., 2019).

Supplemental Table 6. Differences in Cumulative Competitive Employment Outcomes Over 2-Years between Interventions in McGurk et al. (2015) Study (Thinking Skills for Work: TSW, and Supported Employment Only: SE Only) versus Current Dismantling Study (TSW and Cognitive Self-Management: CSM)

|  | 2015 (n = 107) | | Dismantling (n = 203) | | Difference | | |
| --- | --- | --- | --- | --- | --- | --- | --- |
| Employment Measure | *N / M* | % / SD | *N / M* | % / SD | | χ^2^/*Z* | *p* |
|  | TSW | | TSW | |  | |  |
| Any work | 34 | 60.0 | 66 | 66.7 | -1.171 | | .242 |
| Weeks worked | 23.9 | 31.4 | 21.0 | 28.63 | -.820 | | .413 |
| Waged earned ($) | 3,421 | 5,719 | 4,043 | 7,360 | .761 | | .447 |
|  | SE Only | | TSW | |  | |  |
| Any work | 18 | 36.0 | 66 | 66.7 | -5.178 | | <.001 |
| Weeks worked | 9.2 | 19.6 | 21.0 | 28.63 | 3.817 | | <.001 |
| Waged earned ($) | 1,728 | 4,531 | 4,043 | 7,360 | | 2.969 | .003 |
|  | TSW | | CSM | |  | |  |
| Any work | 34 | 60.0 | 73 | 70.2 | -1.811 | | .070 |
| Weeks worked | 23.9 | 31.4 | 24.0 | 29.25 | .028 | | .978 |
| Waged earned ($) | 3,421 | 5,719 | 3,960 | 5,702 | .790 | | .430 |
|  | SE Only | | CSM | |  | |  |
| Any work | 18 | 36.0 | 73 | 70.2 | -5.808 | | <.001 |
| Weeks worked | 9.2 | 19.6 | 24.0 | 29.25 | 4.705 | | <.001 |
| Waged earned ($) | 1,728 | 4,531 | 3,960 | 5,702 | 3.507 | | <.001 |

Notes: *Z* statistic using Mann-Whitney test and χ*^2^* statistic using Walk χ*^2^* test.

All analyses presented are two-tailed tests.


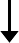
Supplemental Figure 1. Consort Diagram

Assessed for

eligibility (n=758)

Did not meet inclusion criteria (n=343)

Met eligibility but refused to give consent (n=100)

Unable to be contacted (n=64)


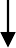


Officially withdrawn from study: [(n=9): CSM (n=2), TSW

(n=7)]: No longer interested in study (n=4); Symptomatic

(n=2); Bereavement (n=1); Surgery (n=1), No longer

interested in work (n=1)

Deceased from study: [(n=4); CSM (n=2), TSW (n=2)]).

*Note that from time of death deceased participants were no*

*longer considered eligible for future assessments.*

Assessed at post-treatment (8 mo.) (n=92)

Not assessed: (n=12)

- Refused (n=1)
- Unable to contact (n=9)
- Deceased (n=1)
- Withdrawal (n=1)

Assessed at 24 months (n=71)

Not assessed: (n=28)

- Refused (n=6)

- Unable to contact (n=13)

- Deceased (n=2)

- Withdrawal (n=7)

Assessed at 16 months (n=67)

Not assessed: (n=32)

- Refused (n=8)

- Unable to contact (n=18)

- Deceased (n=1)

- Withdrawal (n=5)

TSW (n=99)

Exposed to TSW: received ≥6 computer cognitive training sessions (n=62)

Not exposed to TSW: received <6 computer cognitive training sessions (n-37)

Exposed to CSM within this arm: received ≥6 cognitive self-management sessions (n=55)

Not exposed to CSM within this arm: received

<6 cognitive self-management sessions (n=44)

Did not complete baseline (n=17) Failed cognitive screen (n=26)

Consented but ineligible (n=3)

Declined (n=2)

CSM (n=104)

Exposed to CSM: received ≥6 cognitive self-management sessions (n=75)

Not exposed to CSM: received <6 cognitive self-management sessions (n=29)

Not randomized (n=48)

Assessed at 16 months (n=84)

Not assessed: (n=20)

- Refused (n=3)
- Unable to contact (n=14)
- Deceased (n=1)
- Withdrawal (n=2)

Assessed at post-treatment (8 mo.) (n=75)

Not assessed: (n=24)

- Refused (n=9)

- Unable to contact (n=15)

- Deceased (n=0)

- Withdrawal (n=0)

Assessed at 24 months (n=82)

Not assessed: (n=22)

- Refused (n=1)
- Unable to contact (n=17)
- Deceased (n=2)
- Withdrawal (n=2)

Randomized (n=203)

Consented (n=251)

Refused to give consent (n=100):

Not interested in participating (n=35);

Could not commit to weekly sessions

(n=17); Not interested in research

(n=9); Lack of transportation (n=6);

Childcare issues (n=2); Medical

issues (n=1); Vocational rehabilitation

referral (n=1); Did not specify (n=29)

Supplemental Figure 2. Satisfaction with Vocational Services and Finances by Intervention Group Over 2-Year Study Period

Supplemental Figure 3. Competitive Wages Earned by Intervention Group During 6-Month Internals Over 2-Year Study Period
